# Supplementary material for: Uniformly elevated future heat stress in China driven by spatially heterogeneous water vapor changes
Source: Nat Commun. 2024 May 28;15:4522. doi: 10.1038/s41467-024-48895-w (PMC11133461; doi:10.1038/s41467-024-48895-w)
Supplement: Supplementary file 1 — Supplementary Information [file 41467_2024_48895_MOESM1_ESM.pdf]

Supplementary Information for

- **Uniformly elevated future heat stress in China driven by spatially heterogeneous water vapor changes**

Fan Wang *et al.*

*Corresponding author. Email: mmgao2@hkbu.edu.hk and chliu81@ustc.edu.cn

This PDF file includes:

Figs. S1 to S26

Tables S1

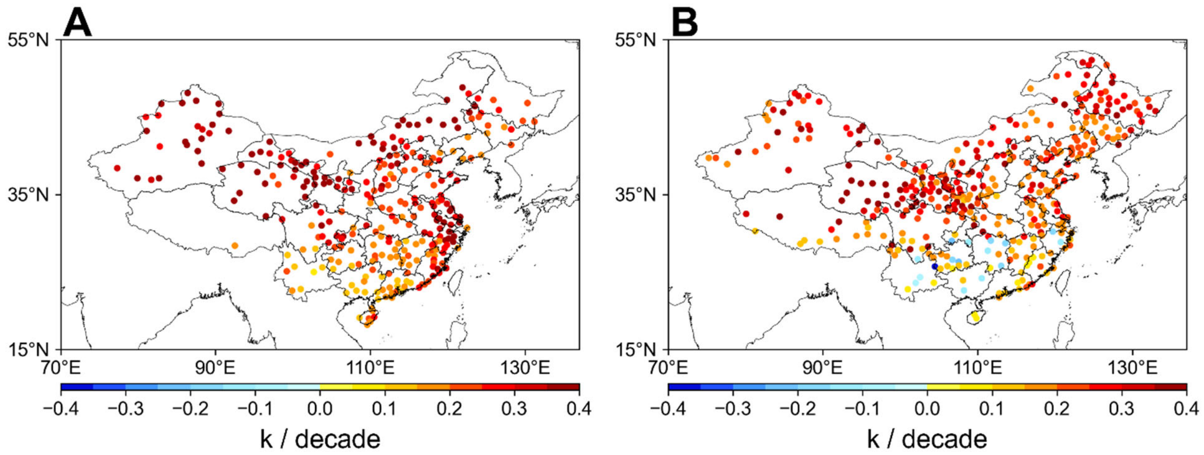

Fig. S1. Wet bulb temperature (T_w) variations. (A) Spatial distribution of T_w variations over 1979-2018 from homogenized data from Argiriou et al (2023) and Li et al (2020). Only sites with significant trend ($P < 0.05$) are displayed. (B) Spatial distribution of T_w variations over 1979-2018 from homogenized data from observations in this study. Only sites with significant trend ($P < 0.05$) are displayed.

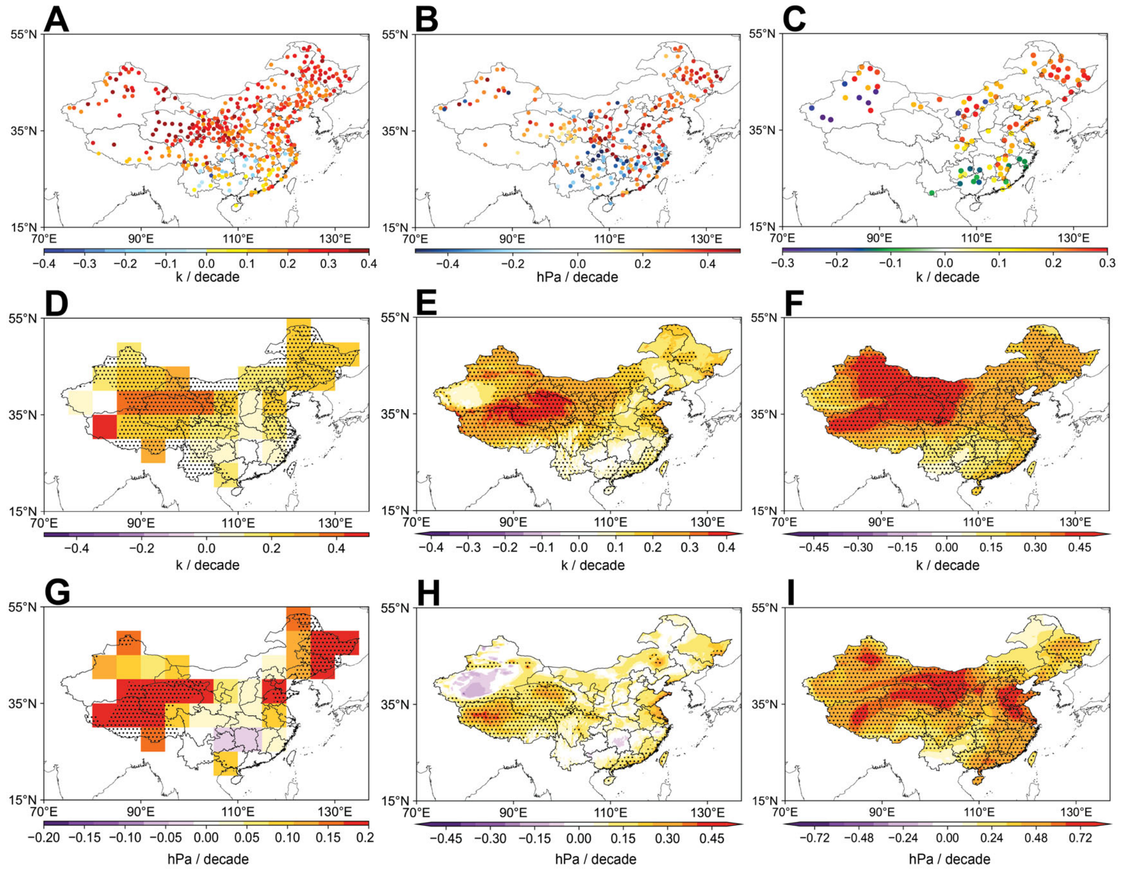

Fig. S2. Wet bulb temperature (T_w) and water vapor (E_a) variations. Spatial distribution of T_w (A) and E_a (B) variations over 1979-2018 from observations. Only sites with significant trend ($P < 0.05$) are displayed. (C) Spatial distribution of T_w variations over 1981-2018 from GSDM-WBT. Only sites with significant trend ($P < 0.05$) are displayed. Spatial distributions of T_w (D) and E_a (G) from HadISDH over 1979-2018. Black dots denote areas with significant trend ($P < 0.05$). Spatial distributions of T_w (E) and E_a (H) from ERA5 over 1979-2018. Black dots denote areas with significant trend ($P < 0.05$). Spatial distributions of T_w (F) and E_a (I) from MERRA2 over 1980-2018. Black dots denote areas with significant trend ($P < 0.05$).

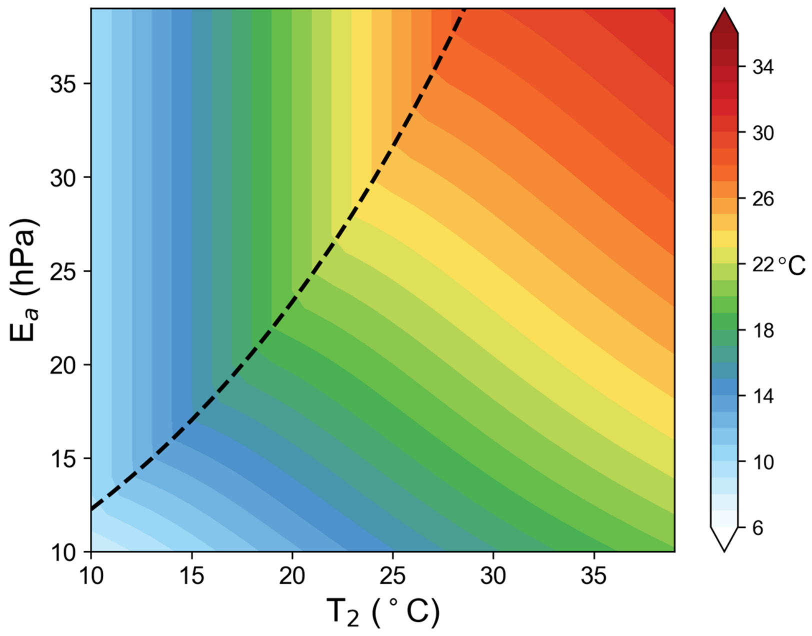

Fig. S3. Sensitivity of wet bulb temperature (T_w) to 2m air temperature (T_2) and water vapor (E_a). The dashed black line indicates saturated water vapor pressure.

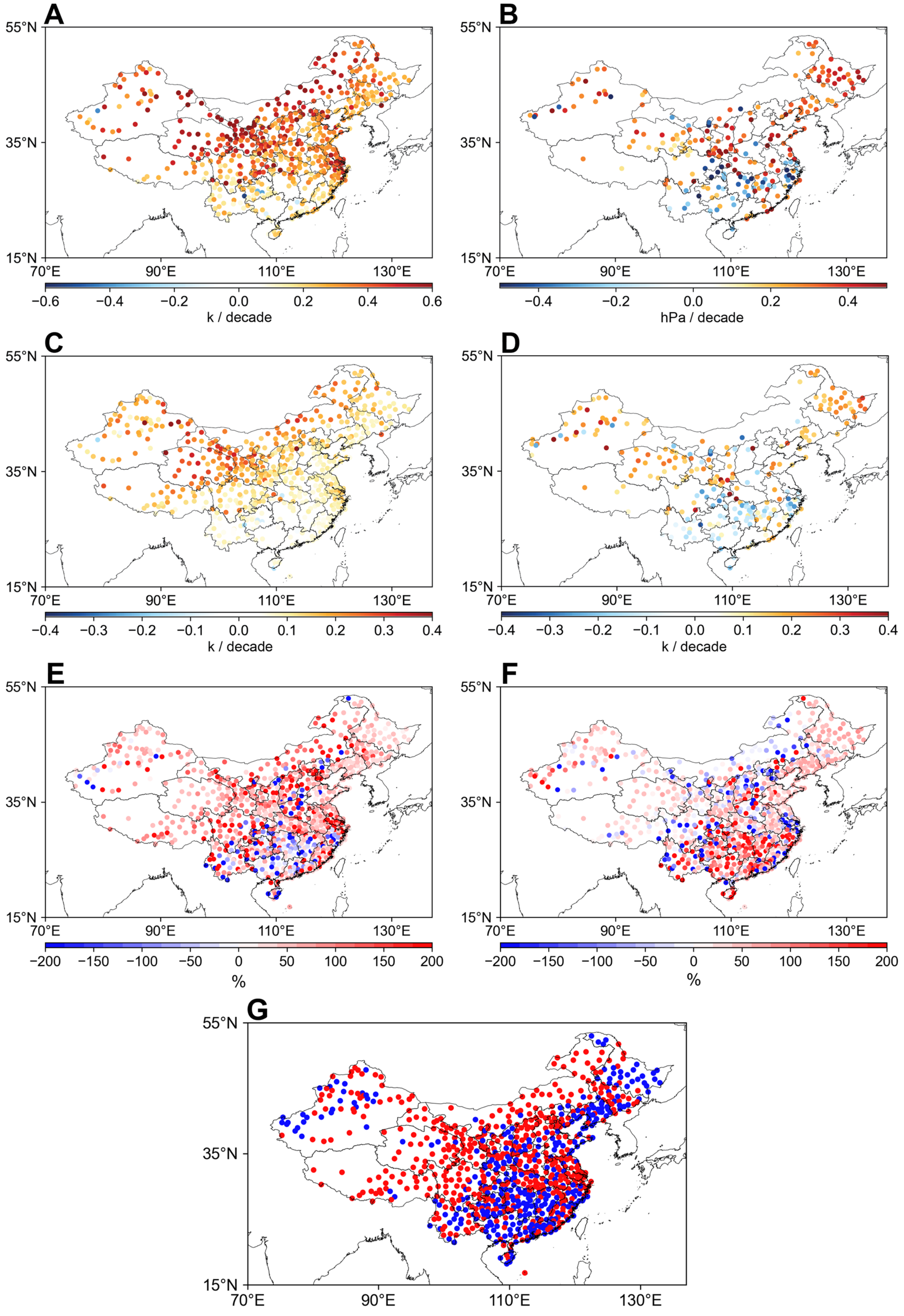
A
55°N
35°N
15°N
70°E
90°E
110°E
130°E
−0.6 −0.4 −0.2 0.0 0.2 0.4 0.6
k / decade
B
55°N
35°N
15°N
70°E
90°E
110°E
130°E
−0.4 −0.2 0.0 0.2 0.4
hPa / decade
C
55°N
35°N
15°N
70°E
90°E
110°E
130°E
−0.4 −0.3 −0.2 −0.1 0.0 0.1 0.2 0.3 0.4
k / decade
D
55°N
35°N
15°N
70°E
90°E
110°E
130°E
−0.4 −0.3 −0.2 −0.1 0.0 0.1 0.2 0.3 0.4
k / decade
E
55°N
35°N
15°N
70°E
90°E
110°E
130°E
−200 −150 −100 −50 0 50 100 150 200
%
F
55°N
35°N
15°N
70°E
90°E
110°E
130°E
−200 −150 −100 −50 0 50 100 150 200
%
G
55°N
35°N
15°N
70°E
90°E
110°E
130°E

Fig. S4. Air temperature (T), water vapor (E_a) and induced wet bulb temperature (T_w) variations. (A) Spatial distribution of T variations during the period from 1979 to 2018. Only sites with significant trend ($P < 0.05$) are displayed. (B) Spatial distribution of E_a variations during the period from 1979 to 2018. Only sites with significant trend ($P < 0.05$) are displayed. (C) Spatial distribution of T induced T_w variations during the period from 1979 to 2018. Only sites with significant trend ($P < 0.05$) are displayed. (D) Spatial distribution of E_a induced T_w variations during the period from 1979 to 2018. Only sites with significant trend ($P < 0.05$) are displayed. Percentage contribution of T_w induced by T (E) and E_a (F) to total T_w changes during the period of 1979-2018. (G) Dominant role of T and E_a on T_w changes. Red indicates the dominant role of T, while blue indicates the dominant role of E_a.

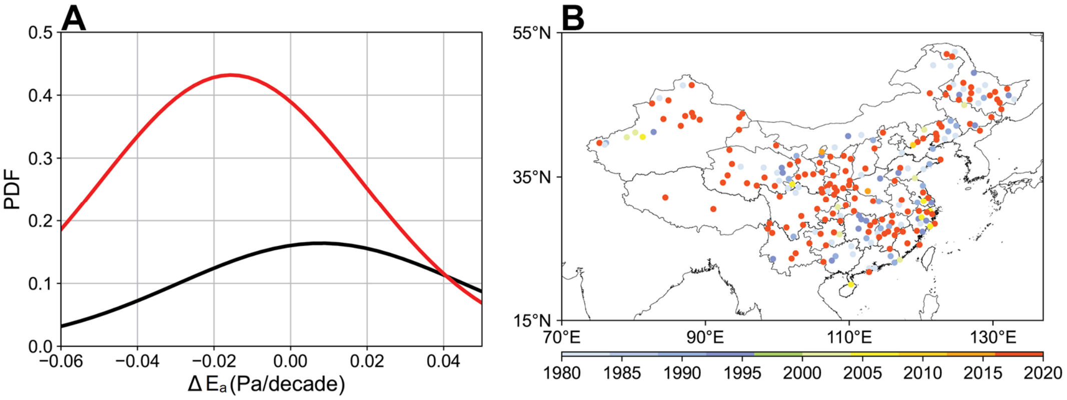

Fig. S5. Land use changes and the impacts on water vapor (E_a). (A) Probability density function (PDF) for changes of E_a in areas with (red line) and without (black line) land use conversion. **(B)** Spatial distribution of temporal evolution of stations' land use transition from natural to impervious urban areas.

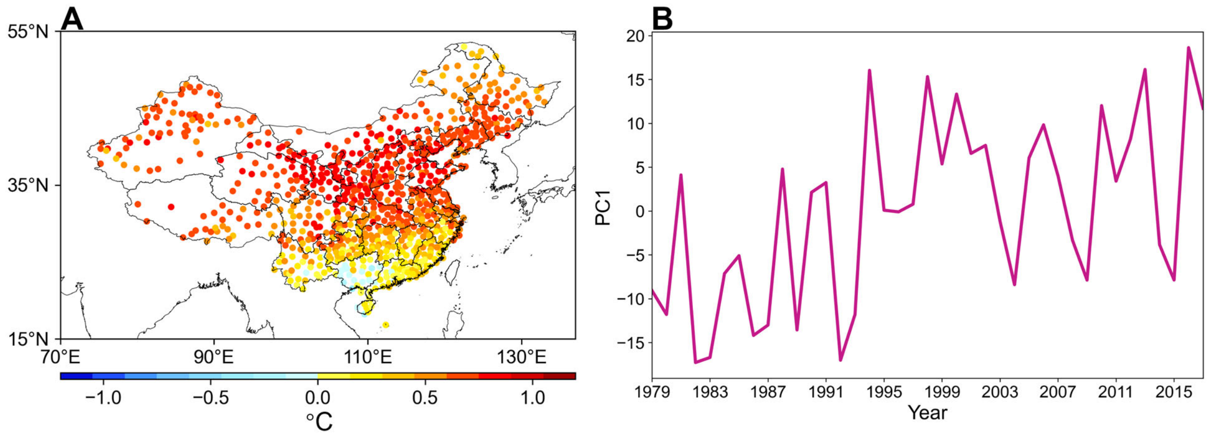

Fig. S6. Empirical orthogonal function (EOF) analysis of wet bulb temperature (T_w). Spatial (A) and temporal variations (B) of the first leading mode inferred by EOF analysis.

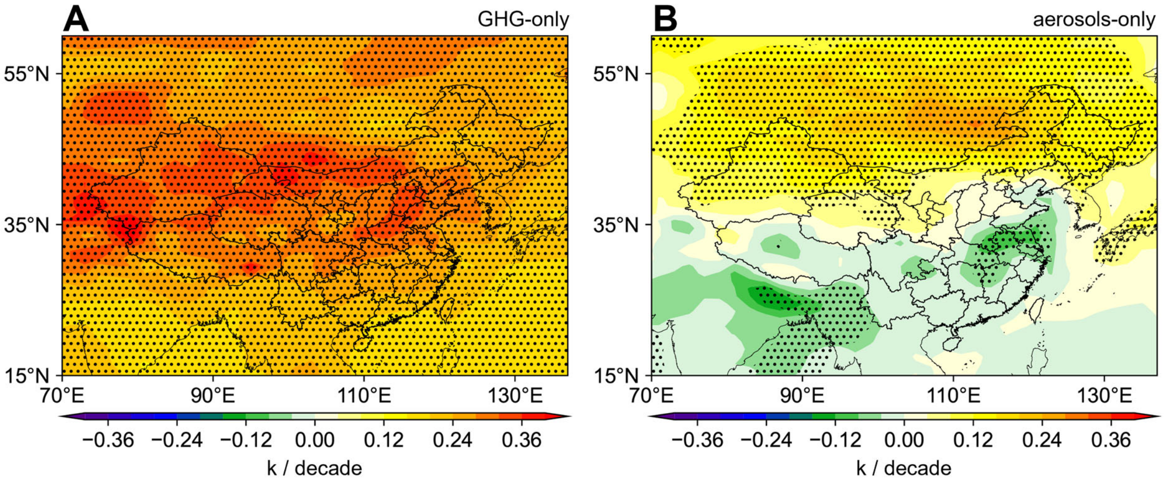

Fig. S7. Global surface temperature variations. Spatial distribution of surface air temperature variations during the period from 1979 to 2014 under GHG-only (A) and aerosols-only (B) forcing conditions. Black dots denote areas with significant trend ($P < 0.05$).

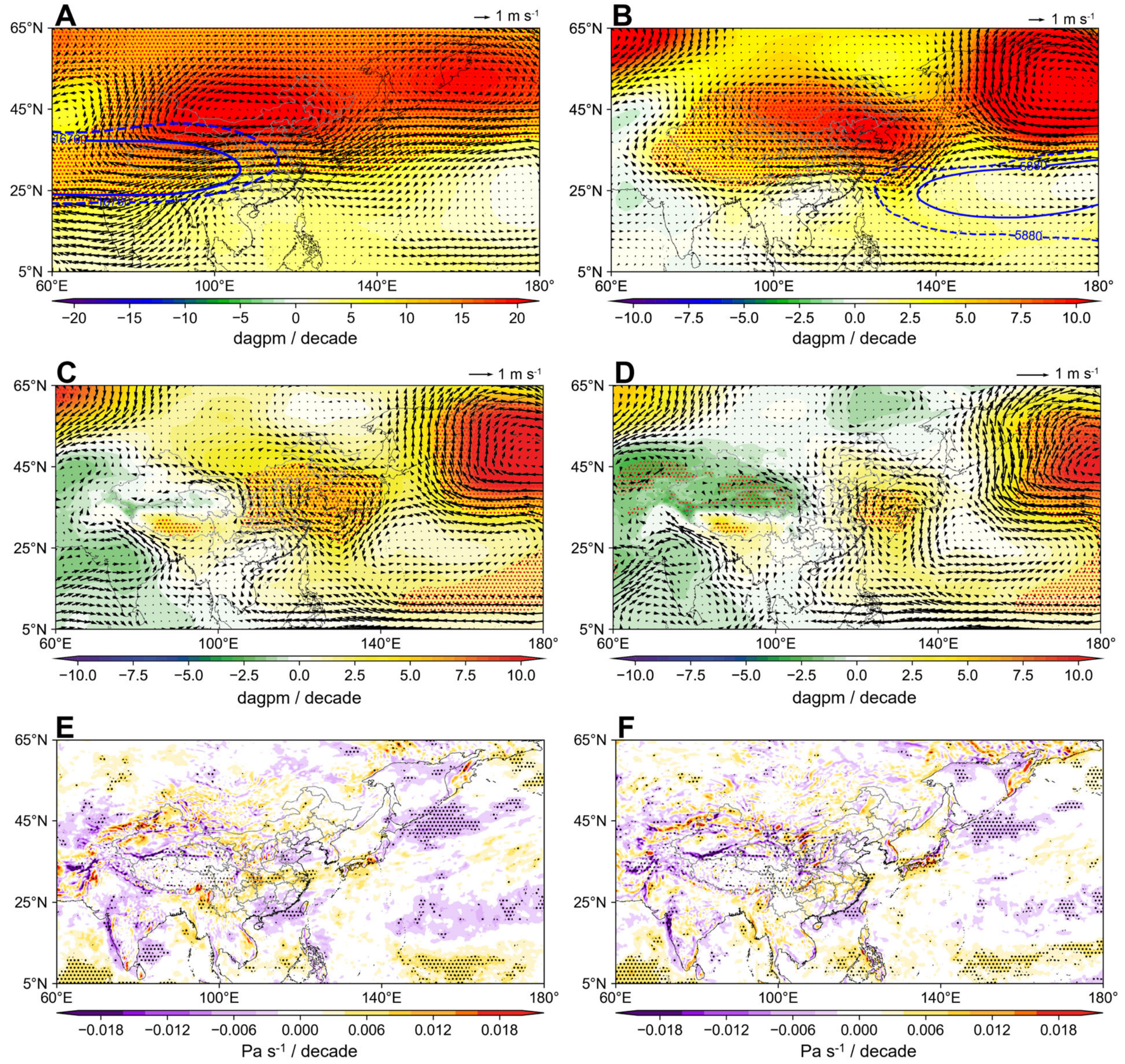

Fig. S8. Regression of atmospheric features on the first leading mode. Regression of geopotential height and circulation at 100 hPa (A), 500 hPa (B), 700 hPa (C) and 850 hPa (D) on the first leading mode. Red dots denote areas with significant correlation ($P < 0.05$). Blues solid lines in A and B indicate climatologically averaged locations of the South Asia high (SAH, represented by 16760-dagpm line) and the western Pacific subtropical high (WPSH, represented by 5880-dagpm line), respectively. Blues dashed lines in A and B indicate varied locations of the SAH and WPSH, respectively. Regression of vertical velocity at 700 hPa (E) and 850 hPa (F) on the first leading mode. Positive values indicate descending motion. Black dots denote areas with significant correlation ($P < 0.05$).

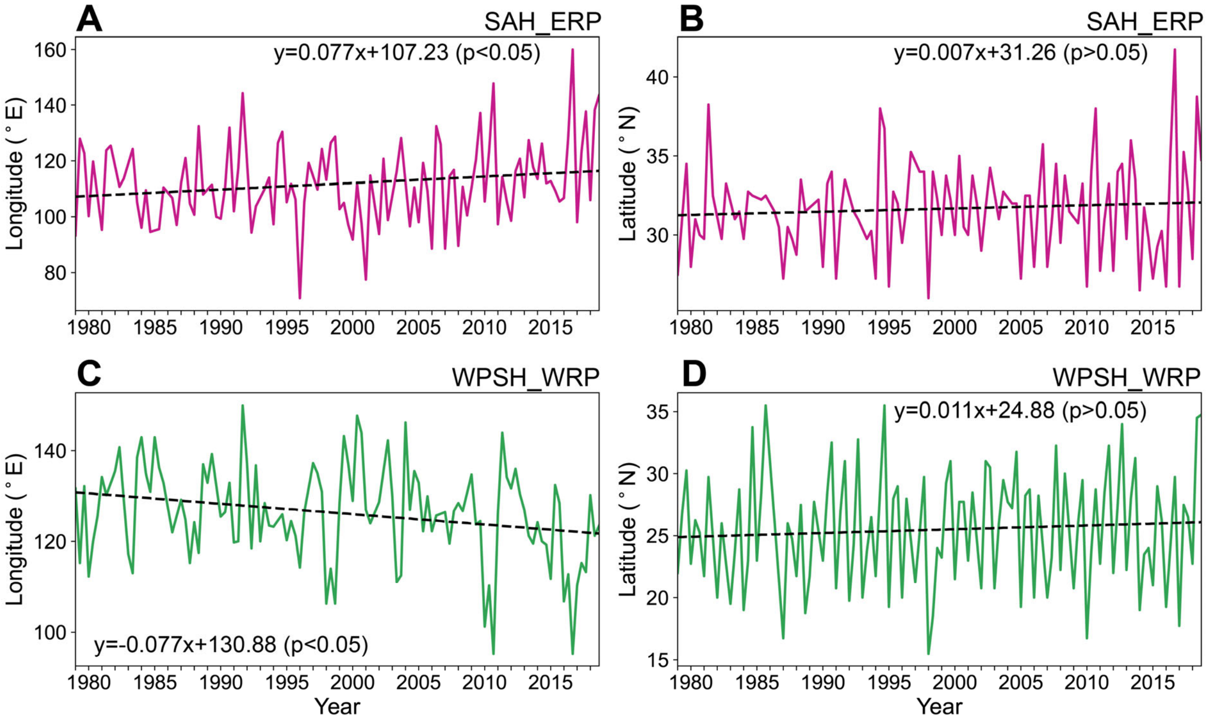

Fig. S9. Variations of locations of the South Asia high (SAH) and the western Pacific subtropical high (WPSH) systems. Time series of monthly longitude (A) and latitude (B) of the eastward ridge point (ERP) of the SAH over the period from 1979 to 2018. Time series of monthly longitude (C) and latitude (D) of the westward ridge point (WRP) of the WPSH over the period from 1979 to 2018.

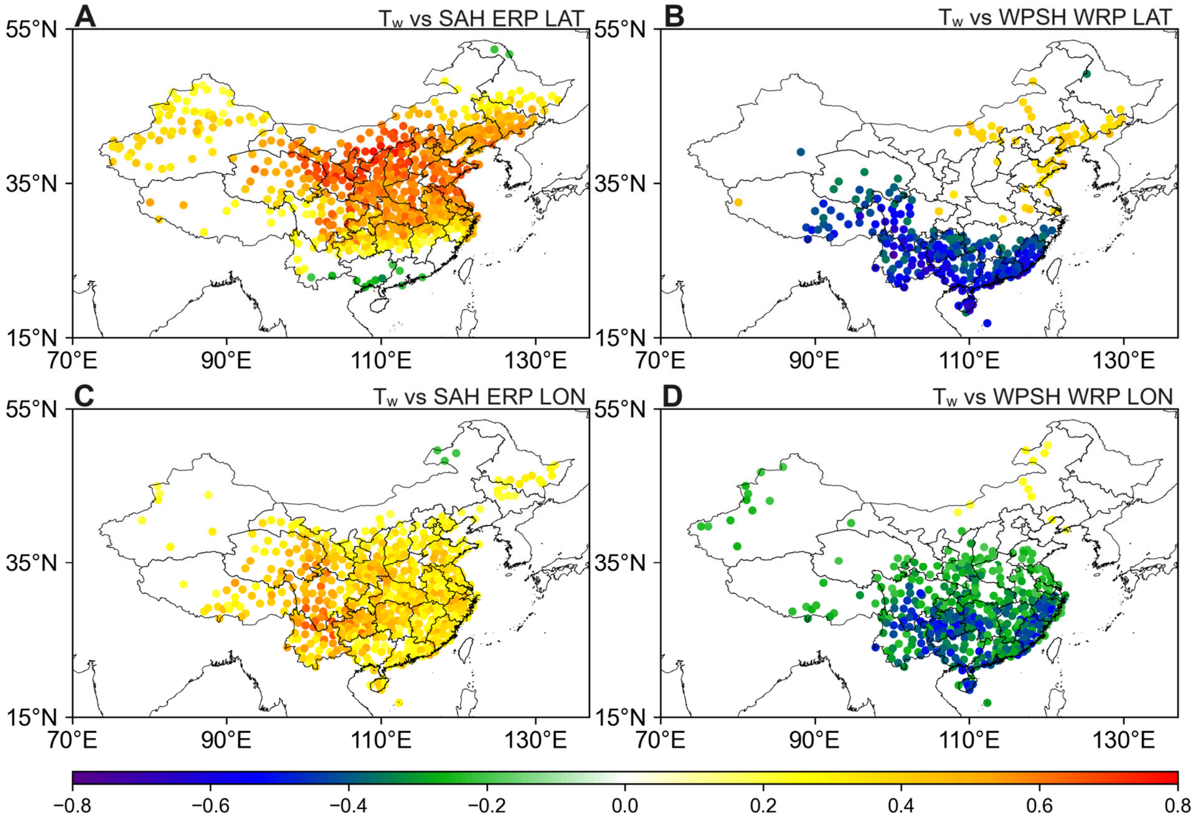

Fig. S10. Correlations of the South Asia high (SAH) and the western Pacific subtropical high (WPSH) with variations of wet bulb temperature (T_w). Correlations of the detrended latitudes of the SAH eastward ridge point (ERP) (A) and WPSH westward ridge point (WRP) (B) with variations of detrended T_w. Correlations of the detrended longitudes of the SAH ERP (C) and WPSH WRP (D) with variations of detrended T_w. Only sites having significant trend with 95% and higher confidence level are displayed.

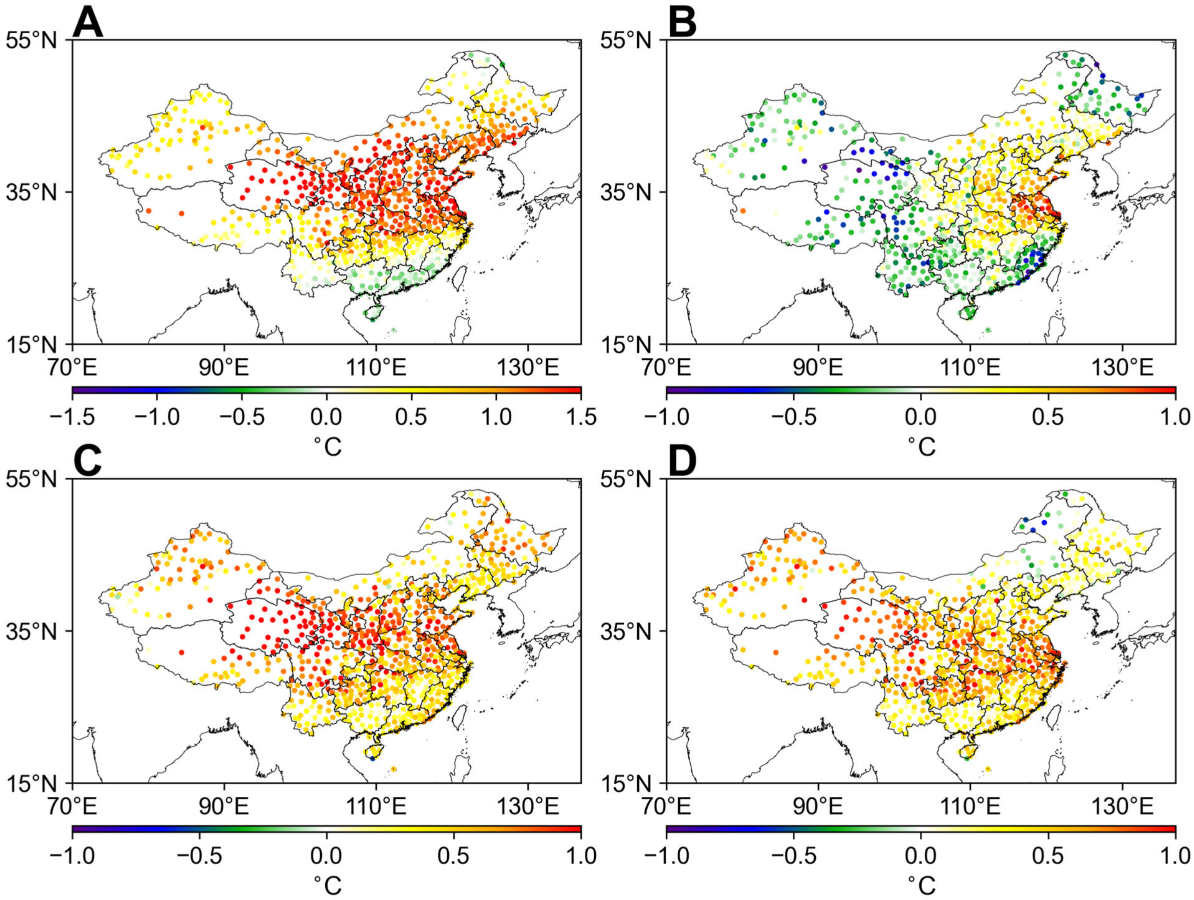

Fig. S11. Composite analysis of wet bulb temperature (T_w). The composite differences of T_w between northward and southward movement of the eastward ridge point (ERP) of the South Asia high (SAH) (A) and the westward ridge point (WRP) of the western Pacific subtropical high (WPSH) (B). The composite differences of T_w between westward and eastward movement of the SAH ERP (C) and WPSH WRP (D).

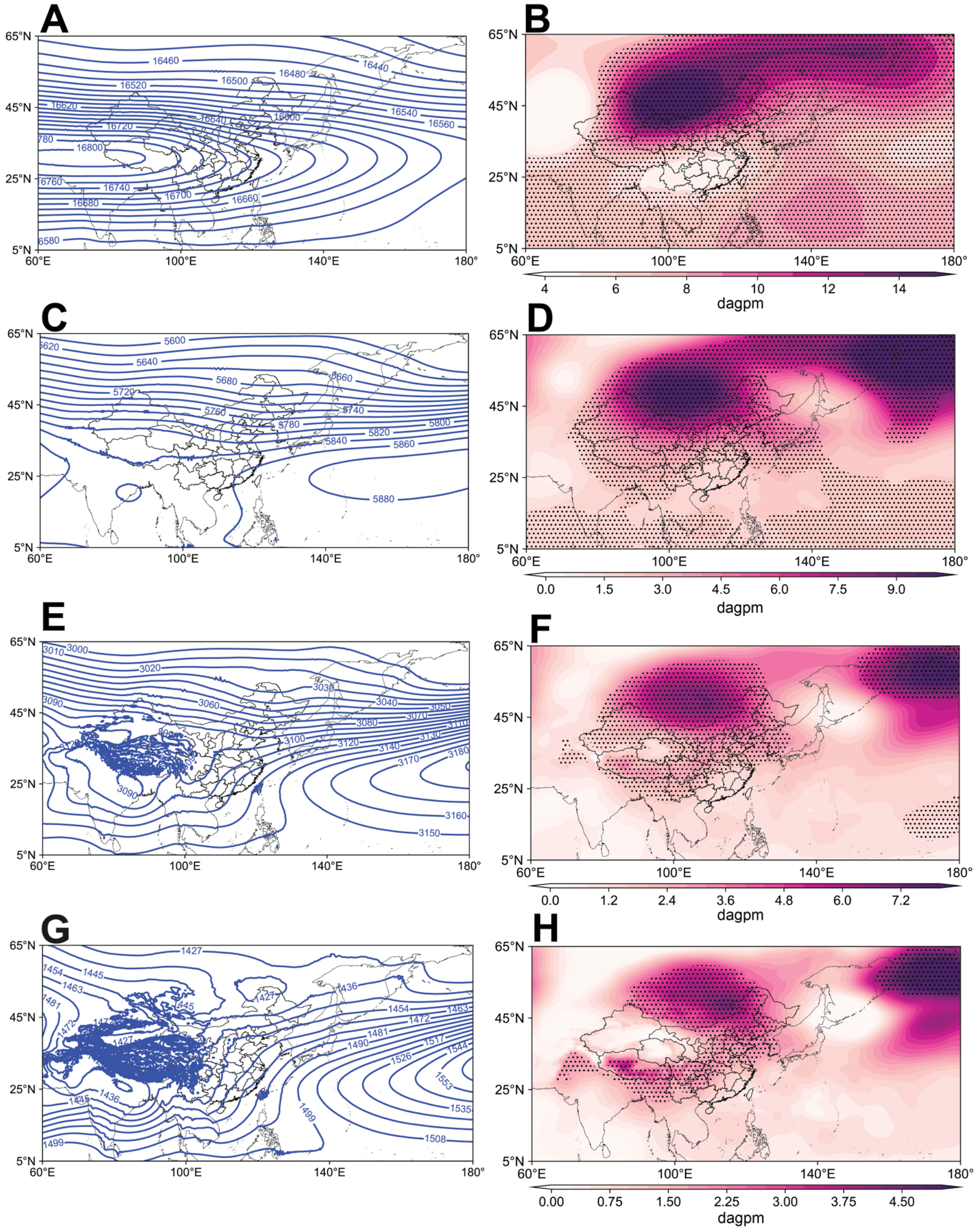

Fig. S12. Atmospheric systems and their evolutions. Spatial distribution of average summertime geopotential height on 100 hPa (A), 500 hPa (C), 700 hPa (E) and 850 hPa (G) over the period from 1979 to 2018. Spatial distribution of geopotential height variations on 100 hPa (B), 500 hPa (D), 700 hPa (F) and 850 hPa (H) over the period from 1979 to 2018. Black dots denote areas with significant trend ($P < 0.05$).

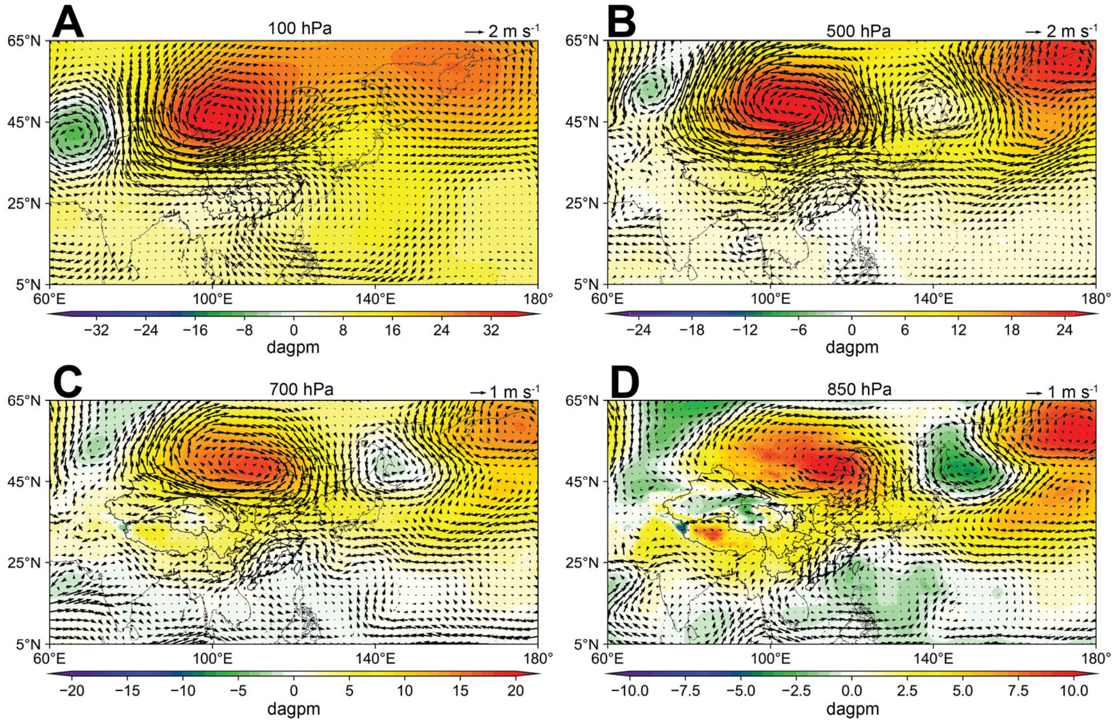

Fig. S13. Changes of atmospheric circulations. Spatial distribution of differences in average summertime geopotential height and atmospheric circulation on 100 hPa (A), 500 hPa (B), 700 hPa (C) and 850 hPa (D) between the period of 1979-1998 and 1999-2018.

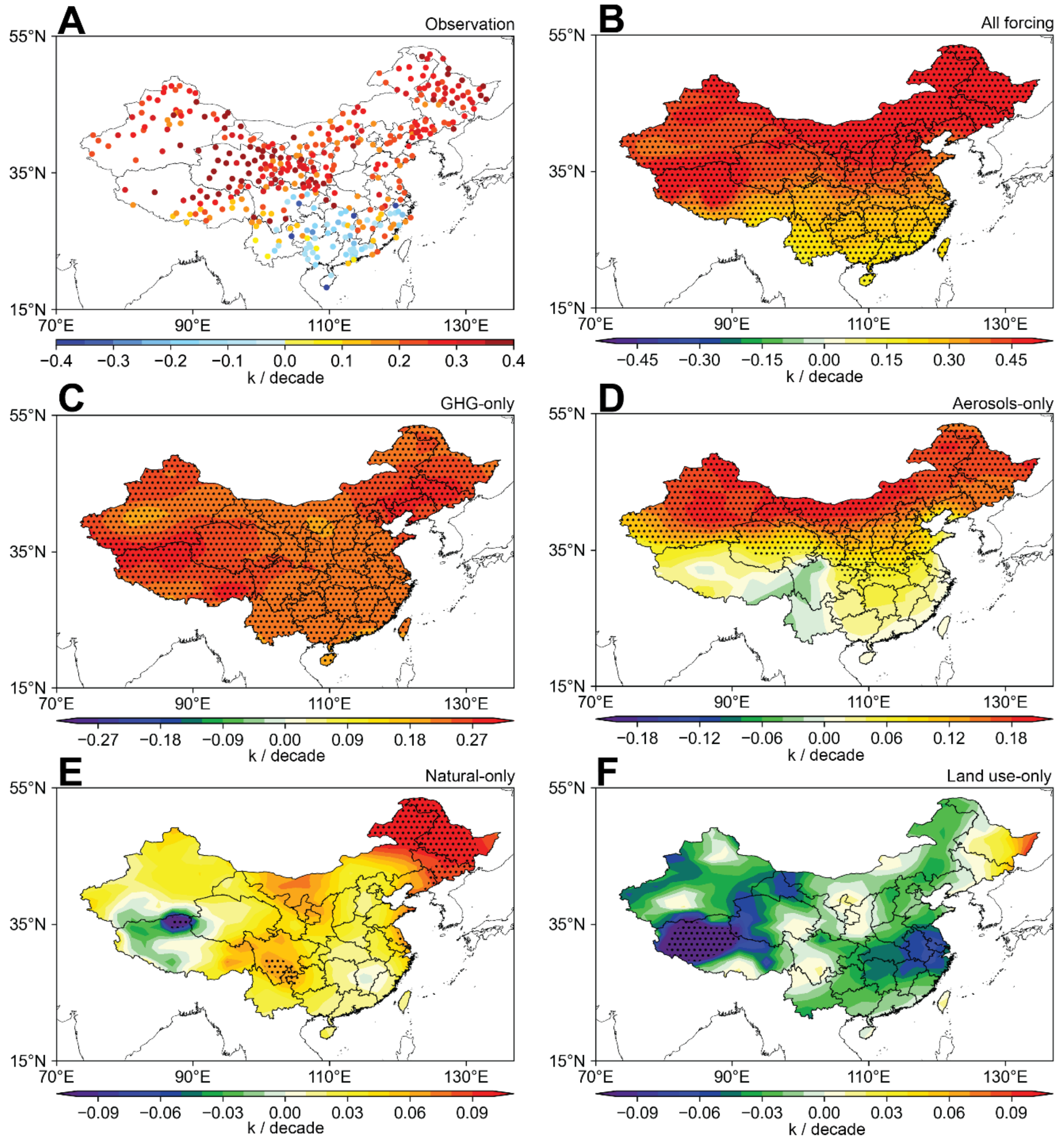

Fig. S14. Observed and simulated variations of wet bulb temperature (T_w). (A) Spatial distribution of observed T_w variations during the period of 1979-2014. Only sites with significant trend ($P < 0.05$) are displayed. **(B)** Spatial distribution of simulated T_w variations under all-forcing conditions during the period of 1979-2014. Black dots denote areas with significant trend ($P < 0.05$). Spatial distribution of simulated T_w variations under GHG-only **(C)**, aerosols-only **(D)**, natural-only **(E)** and land use-only **(F)** forcing conditions during the period of 1979-2014. Black dots denote areas with significant trend ($P < 0.05$).

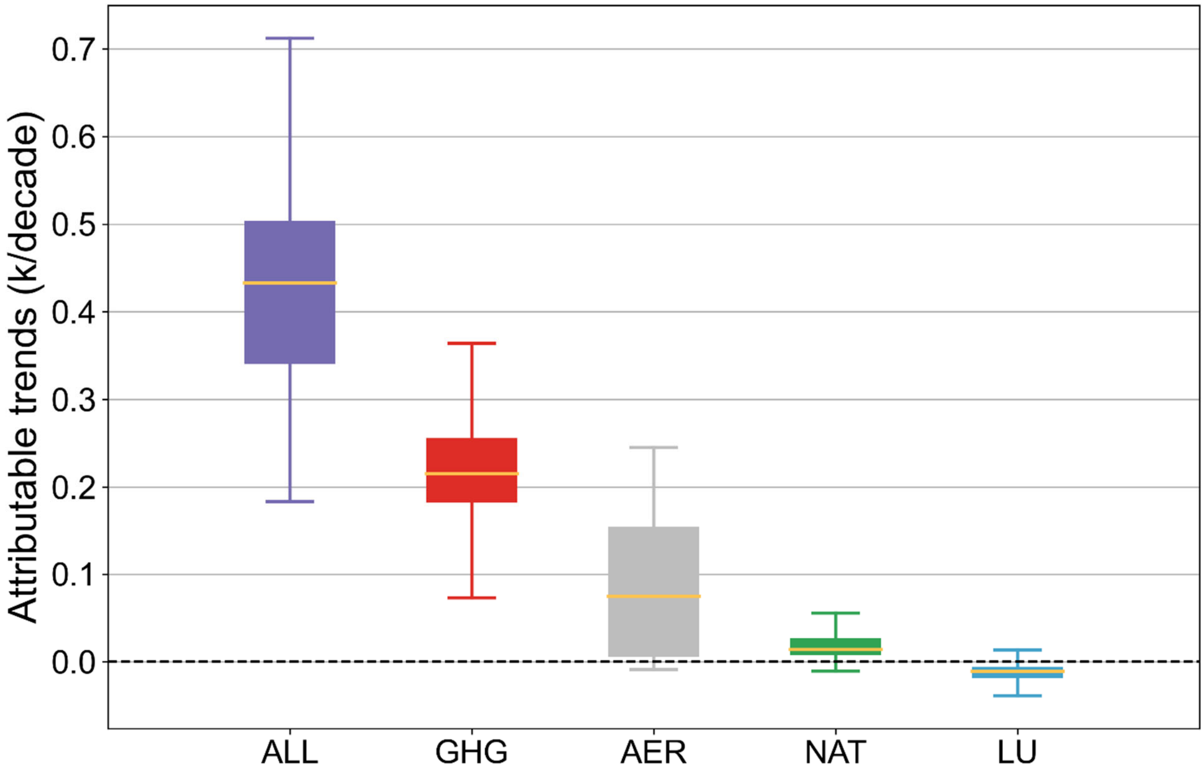

Fig. S15. Attributable variations of wet bulb temperature (T_w) from different contributors. Contributions of individual forcing to variations of T_w caused by all forcings. Yellow lines are median values, box chart values denote median values minus standard deviation, 25% quantile, 75% quantile, and the median value plus standard deviation from bottom to top, respectively.

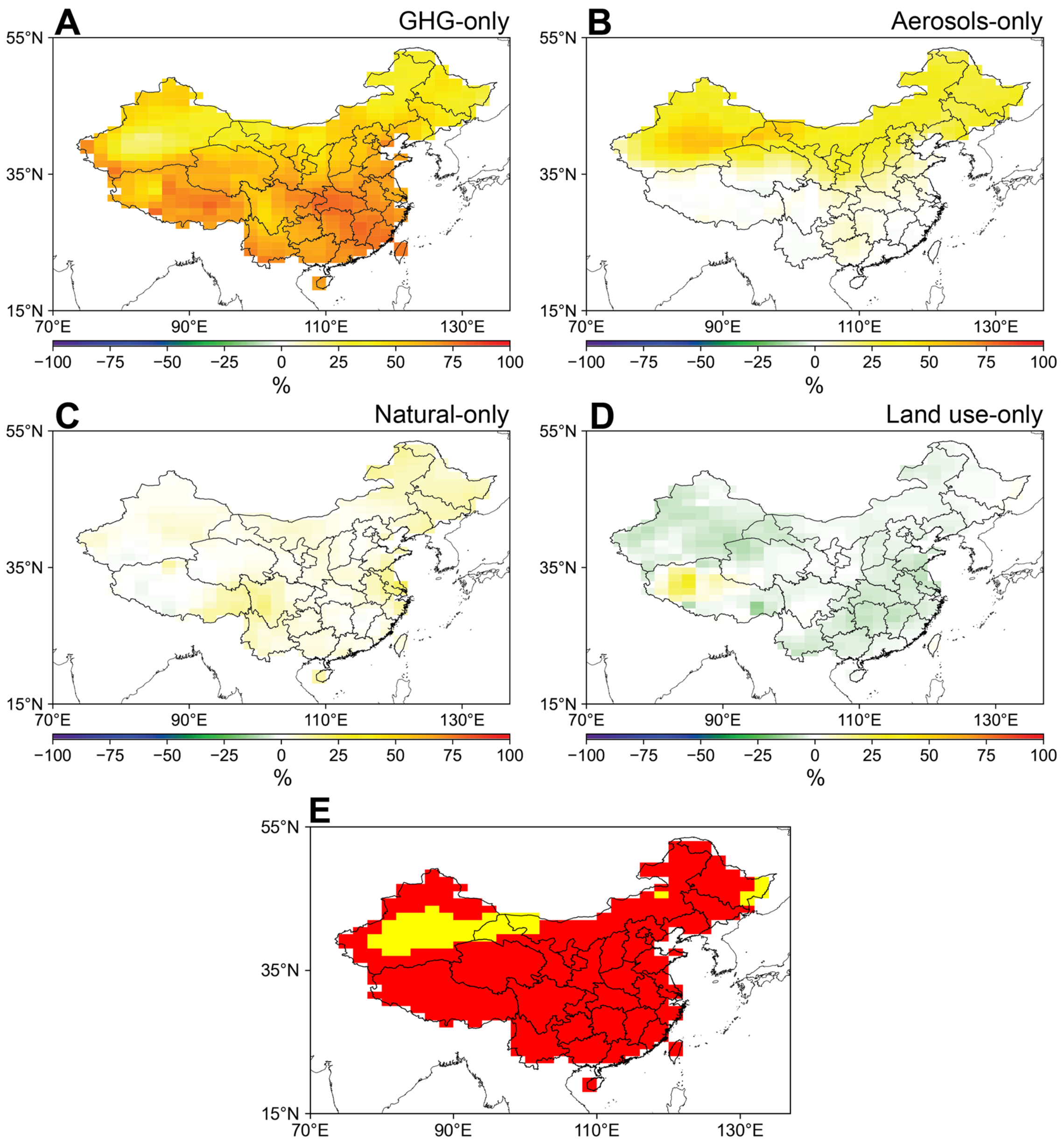

Fig. S16. Percentage contributions of individual forcing to variations of wet bulb temperature (T_w). Percentage contributions of T_w from GHG-only (A), aerosols-only (B), natural-only (C) and land use-only (D) forcing conditions to total T_w variations during the period of 1979-2014. (E) Dominant role of GHG and aerosols on T_w changes. Red indicates the dominant role of GHG, while yellow indicates the dominant role of aerosols.

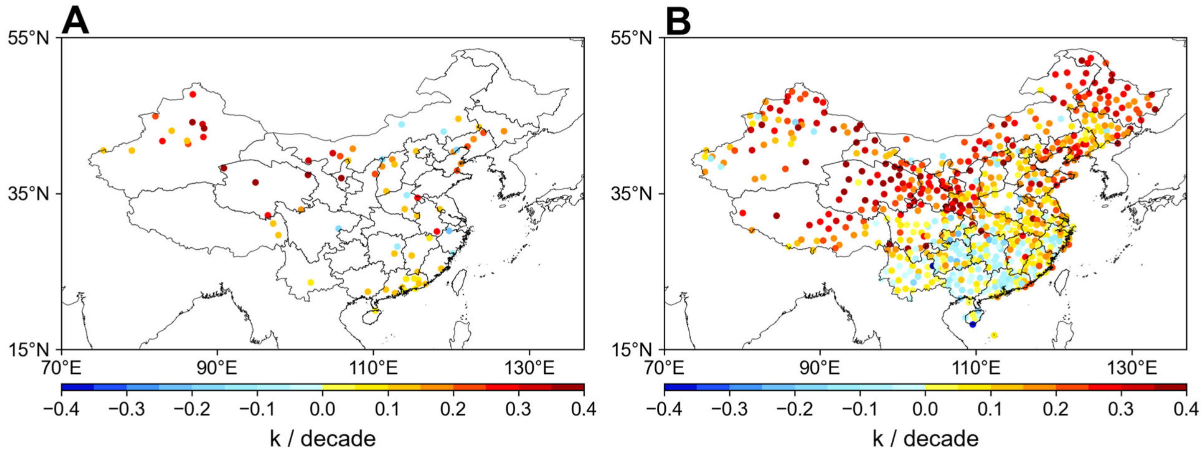

Fig. S17. Empirical decomposition (EMD) analysis on wet bulb temperature (T_w). Spatial distribution of T_w trends caused by natural variabilities (A) and external forcings (B) during the period of 1979-2018.

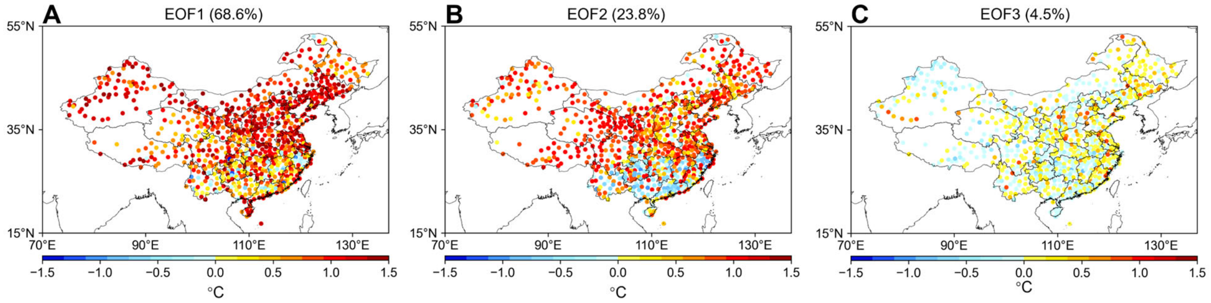

Fig. S18. Empirical orthogonal function (EOF) decomposition of wet bulb temperature (T_w). Spatial patterns of (A) EOF1, (B) EOF2, and (C) EOF3.

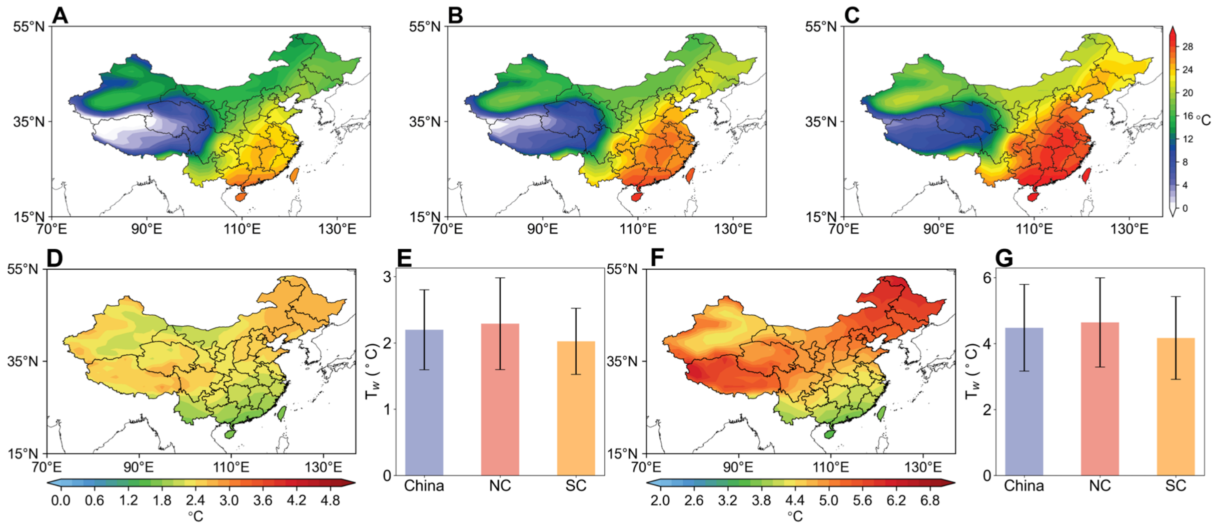

Fig. S19. Spatial distribution of wet bulb temperature (T_w) and its future shifts. Spatial distribution of summertime average T_w over 2010-2014 (A) and over 2096-2100 under the SSP2-4.5 (B) and SSP5-8.5 (C) scenarios from CMIP6 ensemble means. (D) Spatial distribution of differences in summertime average T_w between SSP2-4.5 and Hist from CMIP6 ensemble means. (E) Average changes of T_w in China, northern China (NC) and southern China (SC) between SSP2-4.5 and Hist from ensemble mean of the 12 CMIP6 models listed in Table S2. The error bars show one standard deviation of the multimodal ensemble. (F) Spatial distribution of differences in summertime average T_w between SSP5-8.5 and Hist from CMIP6 ensemble means. (G) Average changes of T_w in China, northern China (NC) and southern China (SC) between SSP5-8.5 and Hist from ensemble mean of the 12 CMIP6 models listed in Table S2. The error bars show one standard deviation of the multi-model ensemble.

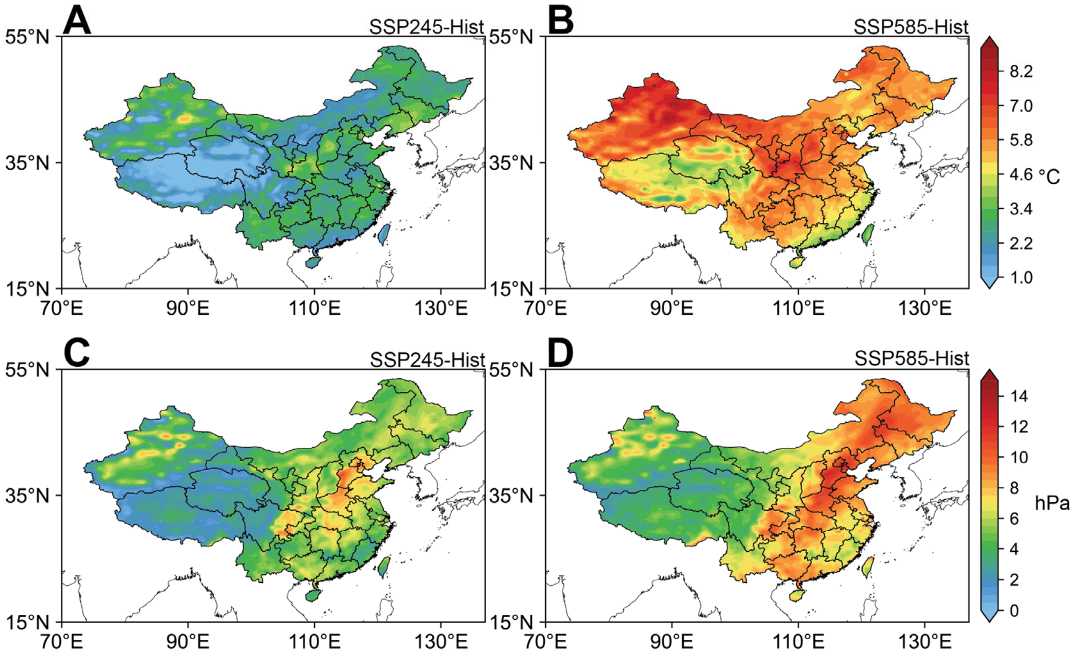

Fig. S20. Future changes of air temperature (T) and water vapor (E_a). Spatial distribution of future changes of T under the SSP245 **(A)** and SSP585 **(B)** scenarios from WRF-Chem simulations. Spatial distribution of future changes of E_a under the SSP245 **(C)** and SSP585 **(D)** scenarios from WRF-Chem simulations.

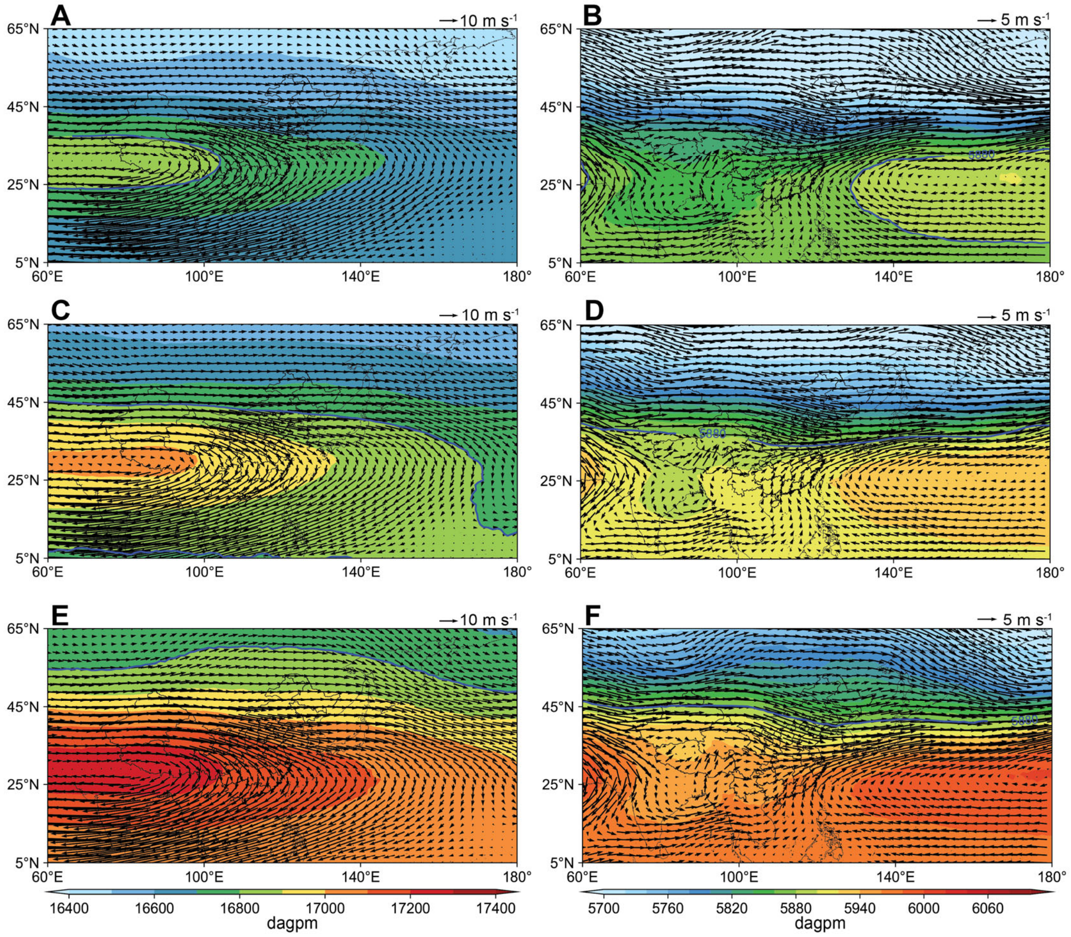

Fig. S21. Historical and future atmospheric circulations. Spatial distribution of average summertime geopotential height and circulation on 100 hPa over the Hist period (2010-2014) **(A)** and future period (1996-2100) under the SSP245 **(C)** and SSP585 **(E)** scenarios from bias corrected CMIP6 global dataset. Spatial distribution of average summertime geopotential height on 500 hPa over the Hist period (2010-2014) **(B)** and future period (1996-2100) under the SSP245 **(D)** and SSP585 **(F)** scenarios from bias corrected CMIP6 global dataset.

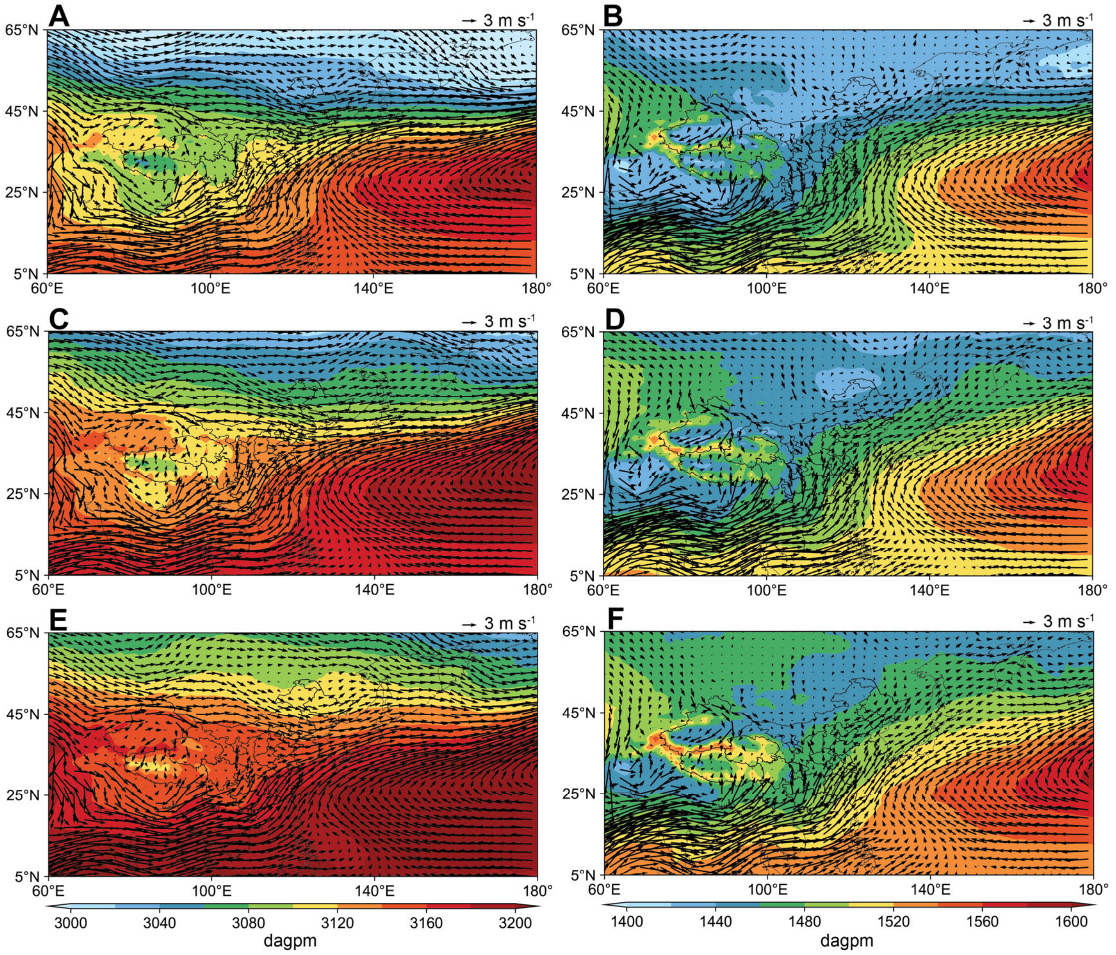

Fig. S22. Historical and future atmospheric circulations. Spatial distribution of average summertime geopotential height and circulation on 700 hPa over the Hist period (2010-2014) (A) and future period (1996-2100) under the SSP2-4.5 (C) and SSP5-8.5 (E) scenarios from bias corrected CMIP6 global dataset. Spatial distribution of average summertime geopotential height on 850 hPa over the Hist period (2010-2014) (B) and future period (1996-2100) under the SSP2-4.5 (D) and SSP5-8.5 (F) scenarios from bias corrected CMIP6 global dataset.

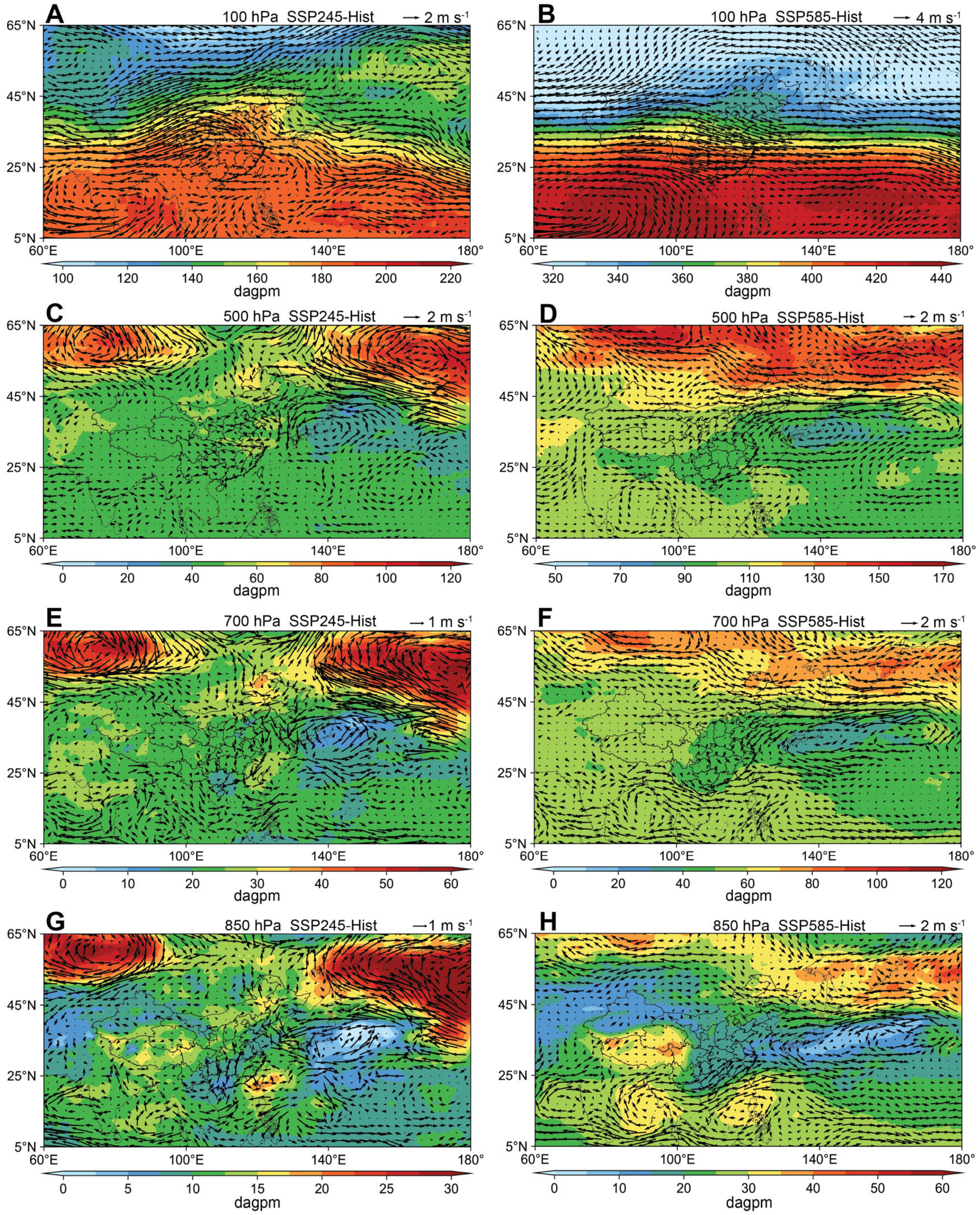

Fig. S23. Future changes of atmospheric circulations. Spatial distribution of future changes of summertime geopotential height and circulation at 100 hPa under the SSP2-4.5 (A) and SSP5-8.5 (B) scenarios from bias corrected CMIP6 global dataset. Spatial distribution of future changes of summertime geopotential height and circulation at 500 hPa under the SSP2-4.5 (C) and SSP5-8.5 (D) scenarios from bias corrected CMIP6 global dataset. Spatial distribution of future changes of

summertime geopotential height and circulation at 700 hPa under the SSP2-4.5 (E) and SSP5-8.5 (F) scenarios from bias corrected CMIP6 global dataset. Spatial distribution of future changes of summertime geopotential height and circulation at 850 hPa under the SSP2-4.5 (G) and SSP5-8.5 (H) scenarios from bias corrected CMIP6 global dataset.

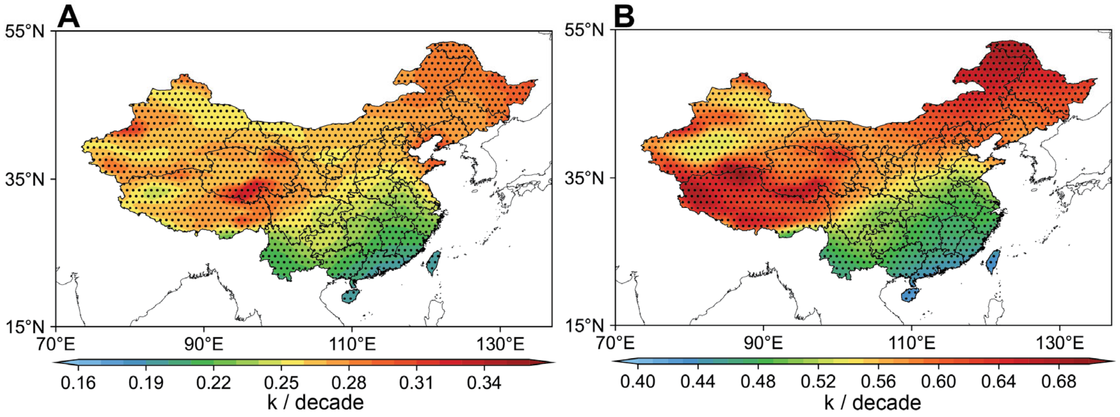

Fig. S24. Future variations of wet bulb temperature (T_w). Spatial distribution of T_w variations under the SSP2-4.5 (A) and SSP5-8.5 (B) scenarios during the period of 2015 to 2100. Black dots denote areas with significant trend ($P < 0.05$).

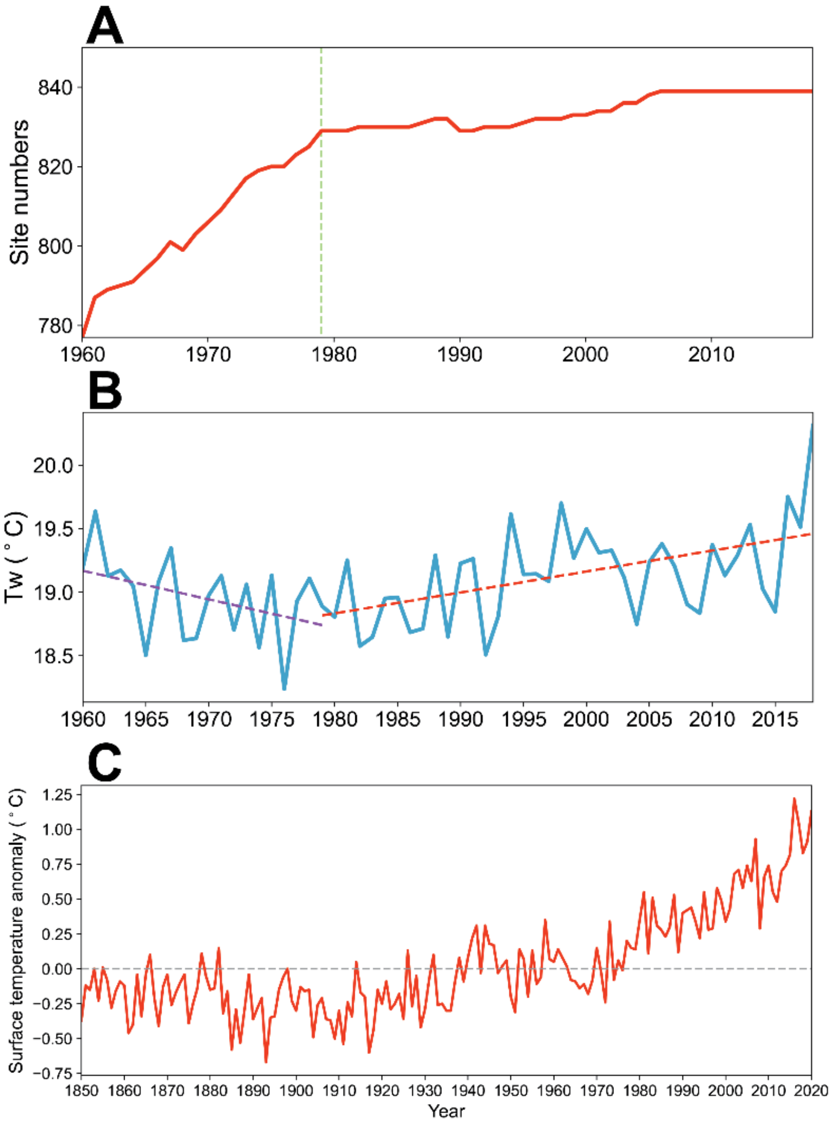

Fig. S25. Site number, wet bulb temperature and surface temperature changes. (A) Number of surface observation stations. (B) Time series of average wet bulb temperature in China from 1960 to 2018. (C) Time series of surface temperature anomaly from 1850 to 2020. Global surface temperature data were obtained from the National Centers for Environmental information (https://www.ncei.noaa.gov/access/monitoring/climate-at-a-glance/global/time-series).

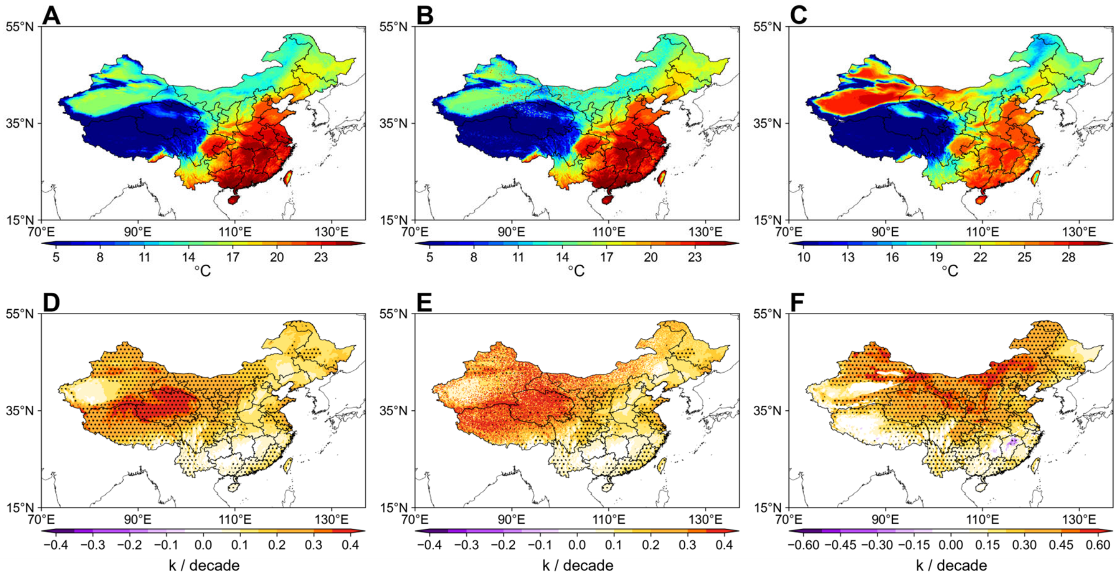

Fig. S26. Historical average and variations of wet bulb temperature (T_w). Spatial distribution of average summertime T_w calculated following method from Stull [1] (adopted in this study) (A), Davies-Jones [2] (B), and Krakauer et al. [3] (C) based on ERA5 data. Spatial distribution of trend of summertime T_w calculated following method from Stull [1] (D), Davies-Jones [2] (E), and Krakauer et al. [3] (F) based on ERA5 data. Black dots denote areas with significant trend ($P < 0.05$).

Table S1. A list of CMIP6 models.

Model Name	Developer	Resolution (lat × lon)	Reference
ACCESS-CM2	Commonwealth Scientific and Industrial Research, Australia	1.25° × 1.875°	Bi et al. [4]
ACCESS-ESM1-5	Commonwealth Scientific and Industrial Research, Australia	1.25° × 1.875°	Ziehn et al. [5]
CESM2	National Center for Atmospheric Research, USA	0.9375° × 1.25°	Gettelman et al. [6]
CanESM5	Canadian Centre for Climate, Canada	2.815° × 2.815°	Swart et al. [7]
GFDL-ESM4	Geophysical Fluid Dynamics Laboratory, USA	1° × 1.25°	Dunne et al. [8]
GISS-E2-1-G	NASA Goddard Institute for Space Studies, USA	2° × 2.5°	Kelley et al. [9]
IPSL-CM6A-LR	Institut Pierre-Simon Laplace, France	1.25° × 2.5°	Boucher et al. [10]
MIROC6	Atmosphere and Ocean Research Institute, Japan	1.40° × 1.40°	Tatebe et al. [11]
MRI-ESM2-0	Meteorological Research Institute, Japan	1.125° × 1.125°	Yukimoto et al. [12]
FGOALS-g3	Institute of Atmospheric Physics, China	2.25° × 2°	Li et al. [13]
CMCC-ESM2	Centro Euro-Mediterraneo sui Cambiamenti Climatici, Italy	1.875° × 1.875°	Lovato et al. [14]

MPI-ESM1-2	Max Planck Institute for Meteorology, Germany	1.241° ×1.875°	Gutjahr et al. [15]

Supplementary references

1 Stull, R. Wet-bulb temperature from relative humidity and air temperature. *Journal of applied meteorology and climatology* **50**, 2267-2269 (2011).

2 Davies-Jones, R. An efficient and accurate method for computing the wet-bulb temperature along pseudoadiabats. *Monthly Weather Review* **136**, 2764-2785 (2008).

3 Krakauer, N. Y., Cook, B. I. & Puma, M. J. Effect of irrigation on humid heat extremes. *Environmental Research Letters* **15**, 094010 (2020).

4 Bi, D. *et al.* Configuration and spin-up of ACCESS-CM2, the new generation Australian community climate and earth system simulator coupled model. *Journal of Southern Hemisphere Earth Systems Science* **70**, 225-251 (2020).

5 Ziehn, T. *et al.* The Australian earth system model: ACCESS-ESM1. 5. *Journal of Southern Hemisphere Earth Systems Science* **70**, 193-214 (2020).

6 Danabasoglu, G. *et al.* The community earth system model version 2 (CESM2). *Journal of Advances in Modeling Earth Systems* **12**, e2019MS001916 (2020).

7 Swart, N. C. *et al.* The Canadian earth system model version 5 (CanESM5. 0.3). *Geoscientific Model Development* **12**, 4823-4873 (2019).

8 Dunne, J. P. *et al.* The GFDL Earth System Model Version 4.1 (GFDL‐ESM 4.1): Overall Coupled Model Description and Simulation Characteristics. *Journal of Advances in Modeling Earth Systems* **12** (2020).

9 Kelley, M. *et al.* GISS‐E2. 1: Configurations and climatology. *Journal of Advances in Modeling Earth Systems* **12**, e2019MS002025 (2020).

10 Boucher, O. *et al.* Presentation and Evaluation of the IPSL‐CM6A‐LR Climate Model. *Journal of Advances in Modeling Earth Systems* **12** (2020).

11 Tatebe, H. *et al.* Description and basic evaluation of simulated mean state, internal variability, and climate sensitivity in MIROC6. *Geoscientific Model Development* **12**, 2727-2765 (2019).

12 Yukimoto, S. *et al.* The Meteorological Research Institute Earth System Model version 2.0, MRI-ESM2. 0: Description and basic evaluation of the physical component. *Journal of the Meteorological Society of Japan. Ser. II* **97**, 931-965 (2019).

13 Li, L. *et al.* The flexible global ocean‐atmosphere‐land system model grid‐point version 3 (FGOALS‐g3): description and evaluation. *Journal of Advances in Modeling Earth Systems* **12**, e2019MS002012 (2020).

14 Lovato, T. *et al.* CMIP6 simulations with the CMCC Earth system model (CMCC‐ESM2). *Journal of Advances in Modeling Earth Systems* **14**, e2021MS002814 (2022).

15 Gutjahr, O. *et al.* Max planck institute earth system model (MPI-ESM1. 2) for the high-resolution model intercomparison project (HighResMIP). *Geoscientific Model Development* **12**, 3241-3281 (2019).
